# Spatial metabolomics: design, pitfalls and data interpretation

**DOI:** 10.1038/s44318-026-00797-x

**Published:** 2026-05-02

**Authors:** Prasad Phapale

**Affiliations:** Environmental Chemistry & Toxicology, Department of Environmental Science, Aarhus University, Roskilde, Denmark

**Keywords:** Metabolism, Methods & Resources

## Abstract

This methods commentary summarises key considerations when mapping metabolites across tissues and organs, reflecting on experimental design, data interpretation and orthogonal validation strategies.

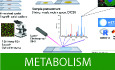

## Teaser

Metabolic heterogeneity across scales is a key driver of biological and physiological processes from tissues down to single cells. Recent advances in mass spectrometry imaging (MSI) allow researchers to map metabolic gradients and centres of activity in intact tissues otherwise masked in bulk analyses. This Comment provides a practical guide to spatial metabolomics, discussing technical challenges and their resolution from experimental design to sample preparation and data analysis.

## Spatial metabolomics illuminates metabolic heterogeneity

Spatial metabolomics using mass spectrometry imaging (MSI) enables untargeted, label-free mapping of metabolite distributions in situ (Najumudeen and Vande Voorde, 2025; Siuzdak, 2023). A key strength of this approach is the ability to capture metabolic heterogeneity within tissues (Samarah et al, 2025), host-microbe interactions (Geier et al, 2020), and cell-type-specific differences that are often invisible to bulk assays(Rappez et al, 2021). As an illustrative example, a recent study mapped metabolic zonation in murine liver, confirming that most metabolites (> 90%) exhibit significant spatial gradients across functional units, consistent with greater energetic demand in e.g., periportal hepatocytes (Samarah et al, 2025). In the small intestine, different TCA metabolites showed opposite spatial enrichment, reflecting distinct nutrient utilization and metabolic division of labor along the crypt-villus axis (Kietzmann, 2017; Samarah et al, 2025). Such results demonstrate how spatial metabolomics offers a powerful new lens on tissue physiology, identifying context-specific substrate utilization and dependent metabolic vulnerabilities.

Spatial metabolomics is equally applicable to disease models, often exposing pathological metabolic reprogramming that is confined to niche regions and cell types (Wang et al, 2022a). An illustrative example comes from acute kidney injury (AKI) after overt recovery from ischemia–reperfusion injury. Spatial metabolomic analysis of post-AKI mouse kidneys revealed persisting regions of metabolic dysfunction in histologically “normal” areas (Rietjens et al, 2025).

To add another layer of complexity, in vivo metabolism is not only compartmentalized at the tissue level but profoundly heterogeneous between cells. Tumors, for instance, exhibit marked metabolic heterogeneity across different regions, which fuels their progression and therapy resistance (Demicco et al, 2024). Until recently, such heterogeneity was inferred indirectly, but advances in spatial metabolomics technologies, including MSI, tracer metabolomics, and single-cell metabolomics, now allow direct mapping of metabolic fluxes inside tumors (Schwaiger-Haber et al, 2023). Several spatial metabolomics technologies are available for researchers and are summarized in Table EV1. However, the following discussion is mainly focused on widely used mass spectrometry imaging modalities, namely MALDI-MSI (Matrix-Assisted Laser Desorption Ionization) and DESI-MSI (Desorption Electrospray Ionization), due to their versatility, relevance to biologists, and wide adoption across research fields (Körber et al, 2025).

A dilemma common to many cutting-edge technologies, the rapid expansion of spatial metabolomics pipelines (summarized in Table EV1) has outpaced the development of standardized experimental and interpretative frameworks. As a result, spatial metabolomics experiments remain vulnerable to technical artifacts, biological misinterpretation, and overgeneralization of spatial patterns. This Comment outlines key experimental and interpretative challenges in spatial metabolomics and aims to offer a concise guide for early adopters of the field, covering individual aspects of the workflow along the way.

### Experimental design: defining spatial context and quality controls

**Figure 1:**
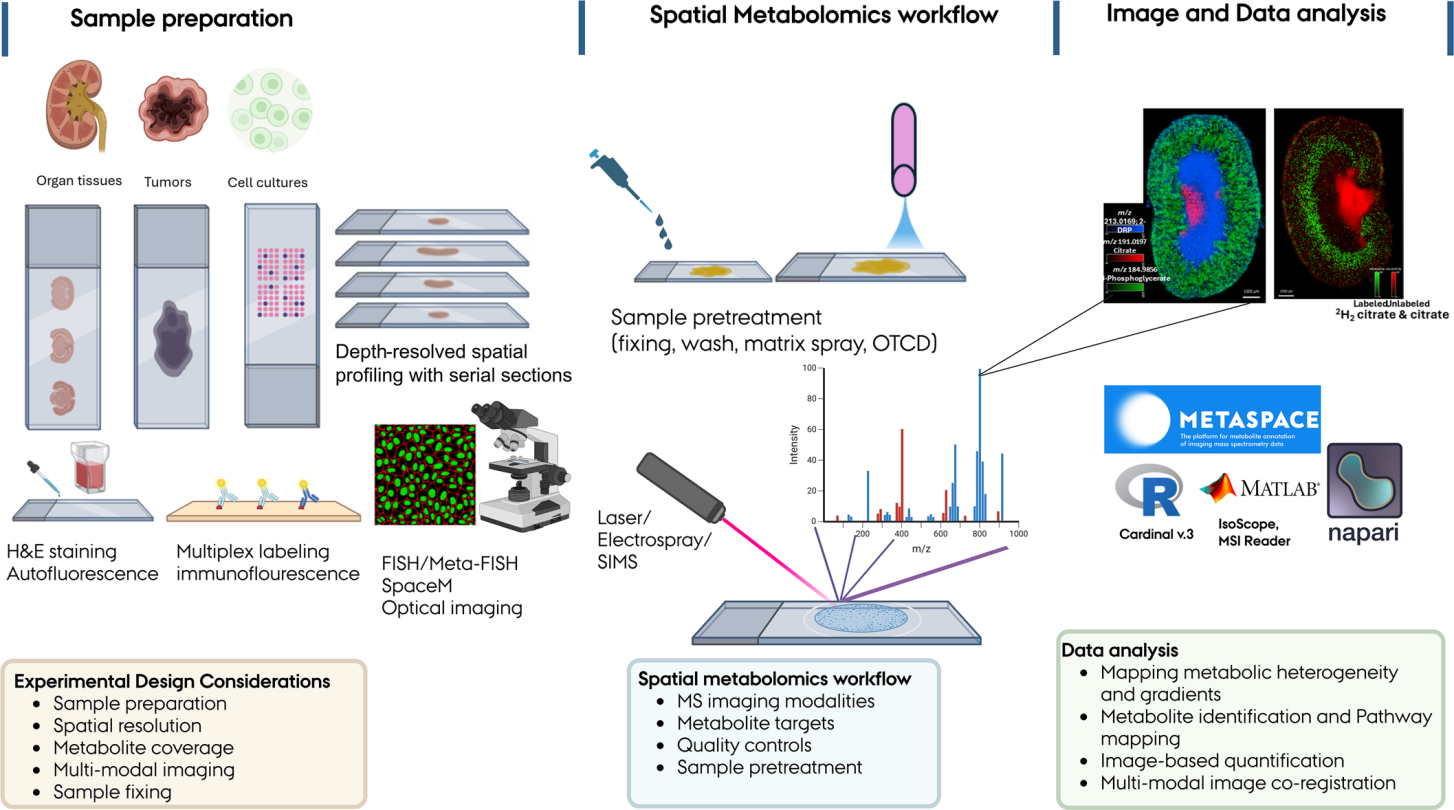
Spatial Metabolomics Workflow. Overview of spatial metabolomics workflow and considerations for experimental design, data acquisition, and interpretation.

Figure 1 illustrates the overall workflow for spatial metabolomics, including sample preparation, instrumentation, and data analysis, highlighting key tools and critical considerations at each stage. A spatial metabolomics experiment should start with a clearly defined biological question that requires spatial resolution and cannot be addressed by bulk analysis. In many cases, bulk metabolomics provides the initial insight that can inform spatial hypotheses tested by imaging approaches. Metabolite coverage in spatial metabolomics is biased by the analytical methods used. It is therefore important to define the metabolites and pathways relevant to the spatial question, as coverage achieved by bulk LC–MS often differs from MALDI-based imaging (Kasarla et al, 2025, 2024). This may include comparisons between defined anatomical regions (e.g., cortex versus medulla in the kidney), metabolic gradients across tissues or tumors, or cell-type- or niche-specific metabolism. The required spatial resolution and molecular coverage should be dictated by this question rather than the technical capability of the imaging platform. Further, batch effects in MSI arise from multiple sources, including day-to-day variations in matrix application (spray density, crystal size), instrument performance drift (laser energy, MS ion optics), environmental factors (humidity, temperature), and matrix degradation during extended acquisition times (Tobias and Hummon, 2020; Huang et al, 2025). These factors need to be tracked with established quality controls and standards specific to the project.

Formulating spatial hypotheses is also essential for defining biological controls and technical replicates, which frequently differ from bulk metabolomics. Spatial controls may include untreated or healthy tissues, sham-operated samples, or internal controls within the same specimen, such as affected versus unaffected regions. In tumor studies, for example, non-tumorous regions identified by histological or immunohistochemical staining can serve as spatially matched controls (Fig. 1). Where feasible, control and experimental samples should be processed, mounted, and analyzed within the same batch or even on the same slide to minimize technical variability. For stable isotope tracing experiments, inclusion of non-tracer controls is essential to account for natural isotopic abundance and background signals. As in bulk metabolomics, biological replication remains critical; multiple independent samples (*n*) per condition are required to ensure that observed spatial patterns are reproducible and statistically meaningful. However, determining an adequate sample size for spatial metabolomics is more complex than for bulk analyses. The appropriate *n* depends on the expected group effect size (within sample and among samples), spatial heterogeneity within and between individual tissue sections (same tissue different depth), the number of metabolites and regions being compared, as well as available resources. Whenever possible, pilot studies are recommended using sample subsets to inform power calculations by estimating within-group variance and effect sizes for key metabolites or regions. Imaging runs should be randomized across conditions where it is possible to mitigate instrument drift, matrix degradation, and sample instability during long acquisition times, which can extend over several hours. However, achieving adequate replication in spatial metabolomics can be challenging due to biological heterogeneity, section-to-section variability, and analytical artifacts such as delocalization or ion suppression, which are difficult to quantify (Ščupáková et al, 2020). Further standardization is required to statistically account for these sources of variability in a spatial context, as addressed by recent advances toward more quantitative spatial metabolomics methods (Wang et al, 2025).

Finally, it is imperative to consider time and instrumentation capacity: a single MSI experiment analyzing one tissue section at 50 μm resolution typically requires 4–8 h and up to 18 h of instrument time for 10–20 μm resolution, compared to ~15–20 min for bulk LC–MS analysis. Such low throughput with required expertise and resource constraints means that spatial metabolomics should be deployed after bulk studies to identify spatial hypotheses worth testing, rather than as a first-line discovery platform.

### Sample collection and metabolic quenching

Organs are metabolically active systems in which metabolite levels can change within seconds to minutes after tissue harvesting. As in bulk metabolomics, rapid tissue collection and effective metabolic quenching are therefore essential to preserve the in vivo metabolic state. Tissues should be collected and flash-frozen immediately, preferably in liquid nitrogen or on dry ice. Any delay in freezing, freeze–thaw cycles, or prolonged handling at room temperature can lead to analyte degradation, enzymatic turnover, and spatial delocalization, ultimately distorting MS imaging results (Moore and Charkoftaki, 2023) as detailed in Table EV2.

Tissue fixation strategies, physical fixation methods, cryo-preservation, heat fixation, and microwave irradiation (summarized in Table EV2)—rapidly inactivate enzymes and are generally compatible with MS imaging (Moore and Charkoftaki, 2023). In contrast, chemical fixation using formalin (FFPE), which is widely employed in histopathology, relies on protein crosslinking or precipitation. However, it can cause substantial analyte loss, chemical modification, and ion suppression, severely compromising metabolite and lipid detection by MS. FFPE tissue represents an invaluable resource of archival collections over decades of clinical specimens linked to patient outcomes, diagnostic data, and longitudinal follow-up. Accessing this biobank material would enable retrospective spatial metabolomic studies that are otherwise difficult with fresh-frozen tissue requirements. Despite challenges, progress has been made in extracting metabolic information from FFPE samples using dewaxing methods, solvent treatments, and targeted stable lipid analysis, as most polar metabolites are lost in treatments (Denti et al, 2022).

Whenever feasible, tissues should be rapidly and uniformly dissected, snap-frozen, and cryosectioned without further treatment. In practice, however, sample preparation must be adapted to the study objectives, tissue type, and downstream analytical requirements, imposing additional constraints. For highly metabolically active organs such as the brain, in vivo metabolic quenching using funnel-freezing approaches has been developed to preserve labile metabolites prior to tissue excision (Mulder et al, 2016). Focused microwave irradiation is used to simultaneously euthanize and fix mouse brain tissue in situ, instantaneously quenching metabolite activities that would otherwise change within seconds after animal sacrifice (Juras et al, 2023). For highly vascularized organs such as the heart, liver, and kidney, transcardial perfusion with cold saline prior to tissue excision is often employed to remove residual blood and reduce overlap with circulating metabolites. This is particularly important for MS imaging, where blood-derived metabolites can obscure tissue-specific spatial patterns.

In all cases, the time between sacrifice and freezing should be minimized. Tissue harvesting, any perfusion steps, and freezing should be carefully coordinated to occur within seconds to, at most, a few minutes. The timing of metabolic quenching is particularly critical in stable isotope tracing experiments, where tissues are often collected at precisely defined time points to capture tracer incorporation. A detailed description of available sample preservation methods is provided in Table EV2 including basic principles, advantages, and limitations for MSI.

### Tissue sectioning

Once tissues are harvested and frozen, sectioning and mounting are key steps that require caution to preserve the spatial integrity of metabolites. Analyses typically require direct cryosectioning of fresh-frozen tissue or prior embedding in MS-compatible supportive media such as carboxymethyl cellulose or gelatin to enable reproducible sectioning. Specialized supportive fixation using conductive carbon tapes (Goodwin et al, 2012) and Kawamoto films (Saigusa et al, 2023) are routinely used for fragile tissues like bone. Such technical complexity of cryosectioning, slide mounting, and subsequent handling in frozen conditions introduces a steep learning curve for metabolomic researchers. Following sectioning, samples are commonly subjected to further processing steps, including slide fixation (discussed above), matrix application (often performed at ambient temperature), and optical imaging or scanning prior to MSI acquisition (Fig. 1). Each of these steps carries a risk of metabolite delocalization or loss if not carefully optimized, underscoring the need for tailored sample-specific optimization protocols. Lastly, a desiccation and vacuum packing step is required before storing or shipping slides at cold temperatures to prevent ice crystals or condensation.

*Where to section?:* A central yet often underappreciated challenge in spatial metabolomics and MS imaging is at which depth and orientation to section tissue. While 3D imaging approaches and volumetric MSI are actively explored, they remain resource-intensive, analytically complex, and impractical for routine use. Consequently, most spatial metabolomics experiments rely on two-dimensional tissue sections, making the choice of section plane and anatomical context critical for biological interpretation.

The sectioning strategy should align with the spatial context and known tissue organization from prior imaging and bulk datasets. In the brain, for example, the choice between coronal, sagittal, or horizontal sections determines which neural circuits, nuclei, or vascular territories are captured. Studies focused on neurotransmitter gradients, ischemic stroke lesions, or region-specific metabolism often require precise anatomical alignment to standardized brain atlases to ensure reproducibility and interpretability across samples (Osetrova et al, 2024). In the heart, short-axis (transverse) versus long-axis (longitudinal) sections capture fundamentally different structural and metabolic features, particularly in models of ischemia–reperfusion injury, myocardial infarction, or hypertrophy. Accurate delineation of ischemic cores, border zones, and remote myocardium requires careful coordination between the sectioning strategy and the known disease pathophysiology. Although the liver is often perceived as structurally homogeneous, it exhibits pronounced metabolic zonation along the porto–central axis (Kietzmann, 2017). Spatial gradients spanning periportal, midzonal, and pericentral regions reflect differential oxygen tension, nutrient availability, and enzyme expression. These considerations are especially important in disease models such as non-alcoholic fatty liver disease (NAFLD), steatohepatitis, or drug-induced liver injury, where zonation patterns may be distorted or selectively amplified. In the kidney, spatial organization is comparatively well defined, with distinct cortical, medullary, and papillary compartments. While this can simplify region-of-interest selection, metabolic gradients along the corticomedullary axis still require consistent sectioning depth and orientation. Across tissues, effective region selection rarely relies on MSI data alone. Instead, it typically requires integration with orthogonal modalities such as hematoxylin and eosin (H&E) staining, immunohistochemistry (IHC) or fluorescence imaging performed on the same or adjacent sections (Fig. 1). Importantly, this process benefits from early collaboration with pathologists and clinicians, ideally during experimental design.

Analyzing tumor tissues often comes with added complexity due to intra-tumoral heterogeneity, including gradients in hypoxia, proliferation, necrosis, immune infiltration, and stromal composition, varies not only within a single section but also across serial sections (Lin et al, 2017). As a result, identifying representative regions for spatial metabolomics often necessitates multiple adjacent or serial sections, combined with other imaging modalities. Taken together, section placement is not a technical but a biological decision that fundamentally shapes spatial metabolomics outcomes; explicit reporting of section orientation and anatomical landmarks should therefore be considered best practice.

### Sample pre-treatments to enhance metabolite imaging

Tissue sections often require pre-treatments prior to MS imaging to improve metabolite detectability and coverage. These may include exposure to buffers, organic solvents, staining reagents, tissue chemical derivatization reagents (OTCD), and MALDI matrix application. In contrast to solvent-based metabolite extraction for bulk analysis, MSI requires metabolites to remain in situ within tissue sections, limiting enrichment of metabolites. Consequently, low-abundance metabolites, including many signaling molecules, intermediates, and regulatory metabolites, are often undetectable or suppressed by more abundant lipids and matrix ions, particularly in the low *m/z* range (< 500 Da). While OTCD and matrix selection can partially mitigate this limitation for specific compound classes, MSI inherently favors the detection of abundant metabolites and lipids with limited coverage of the low-abundance metabolome compared to bulk approaches (Smith et al, 2025; Kasarla et al, 2025).

**Figure 2:**
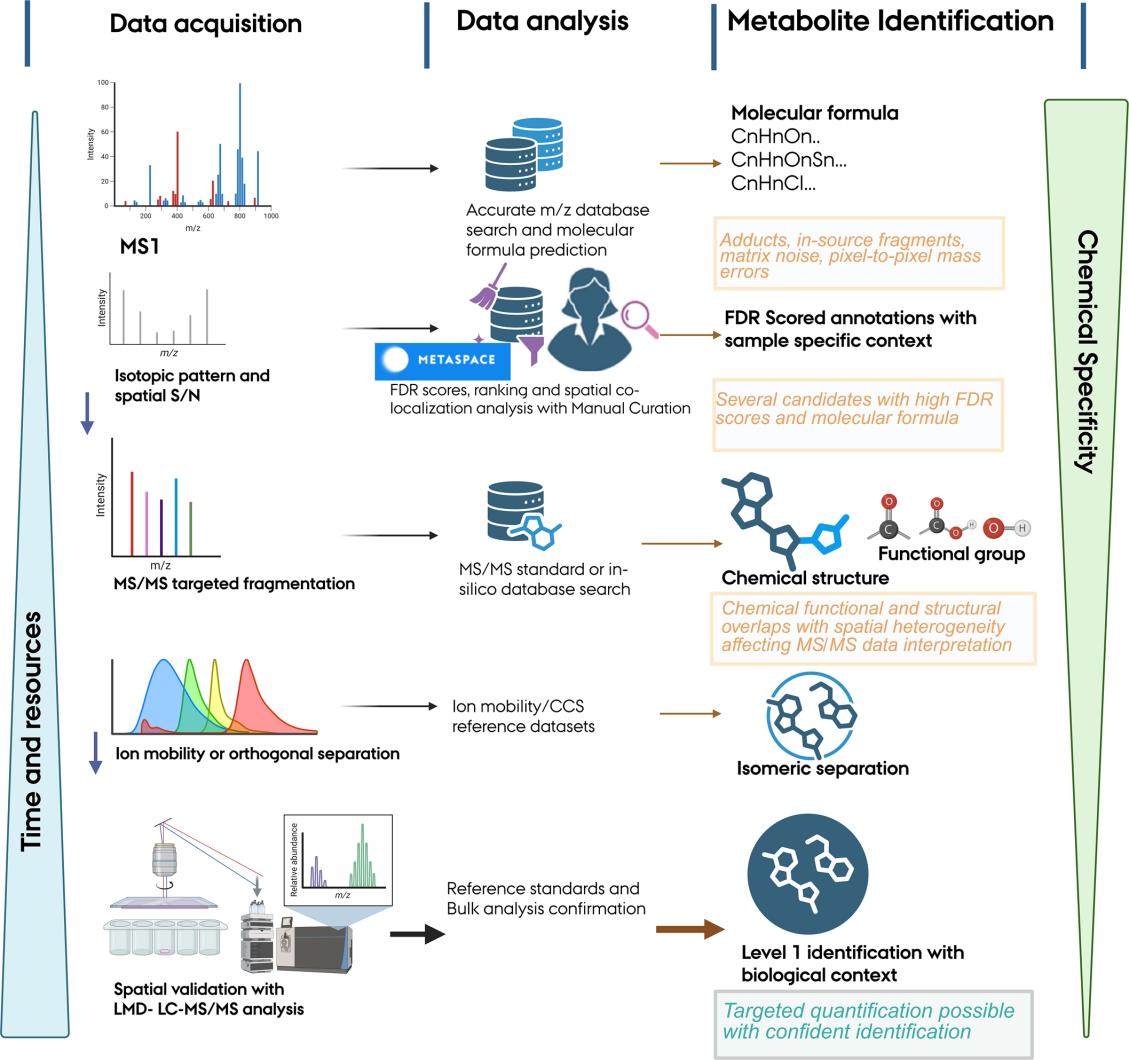
Integrated MS imaging strategy for metabolite identification. Data acquisition approaches, annotation platforms, and validation methods with corresponding trade-off of time/resource requirements with identification confidence and chemical specificity achieved.

Solvent treatments can substantially enhance the detection of specific metabolite classes by removing other competing biomolecules; they also introduce risks of metabolite loss, delocalization, or chemical bias, necessitating careful optimization and validation of protocols. Solvent-based pre-treatments are commonly used to improve the ionization of poorly detected metabolites. For example, acidic methanol or basic hexane washes can enhance the detection of small, polar metabolites by reducing ion suppression and removing interfering lipids or salts (Kasarla et al, 2025; Lu et al, 2023). To image metabolite classes that are poorly detected OTCD and reactive matrix (Zhou et al, 2021) strategies have been developed to selectively modify functional groups and enhance ionization efficiency. While effective for challenging but metabolically important compounds such as carboxylic acids, these approaches increase workflow complexity and may introduce spatial perturbations. Researchers have addressed this by performing spatial validation experiments (Fig. 2) using laser microdissection to cut out specific regions of the tissue and then analyzing those by LC–MS to confirm localization post treatment (Kasarla et al, 2025).

### Multimodal imaging and spatial validation

Spatial metabolomics cannot fully address biological questions and is often required to integrate with other imaging modalities to put the metabolic data in context. One key practice is co-registering MSI data with histological images (H&E or IHC) using adjacent or the same tissue sections those used for MSI. Aligning the MS, autofluorescence, or H&E-stained images helps identify which anatomical structures correspond to metabolic hotspots(Spangenberg et al, 2025). Pre-MSI scanning of tissue sections can be used to get a high-resolution optical image or autofluorescence, IR, or Raman imaging to visualize anatomical and molecular structures. Importantly, this can be done prior to matrix or solvent application, taking advantage of the tissue’s natural autofluorescence (e.g., from molecules such as collagen, NADH, and lipofuscins) to provide contrast between different regions (Patterson et al, 2018). Beyond correlation with histology, spatial metabolomics can be combined with other metabolic imaging modalities as MRI or micro-CT on the intact sample (Ntziachristos et al, 2019). Figure 2 illustrates challenges of the metabolite identification process and spatial validation with orthogonal technologies.

### Challenges in data interpretation

Running spatial metabolomics frequently constitutes substantial challenges in data interpretation, arising from incomplete metabolome coverage, limited quantification, data complexity, and dependence on orthogonal measurements. A fundamental limitation is restricted metabolome coverage inherent to specific imaging methods; a large-scale evaluation of 24 commonly used MSI workflows demonstrated that none could detect all 172 tested metabolites, with coverage strongly influenced by matrix choice, ionization mode, and instrument settings (Saharuka et al, 2024). Similarly, quantitative inter-laboratory and longitudinal batch comparisons routinely done in bulk metabolomics are often not feasible in spatial workflows. As illustrated in Fig. 2, MS imaging data is acquired in MS1 mode and usually without set quality controls or internal standards. These practices are changing considerably to make MS imaging more quantitative (Tobias and Hummon, 2020; Wang et al, 2025) and community resources integrated with platforms such as METASPACE and napari (Saw et al, 2025) now catalog protocol-dependent detectability and can guide parameter selection and metabolite annotation(Wadie et al, 2024; Smith et al, 2025).

Adduct formation and in-source fragmentation in MALDI mode are more pronounced and variable than in LC–MS, however, remains insufficiently characterized which can lead to miss annotations. Further, metabolite identification in spatial metabolomics is limited by the lack of routine MS/MS acquisition, and additional targeted experiments on-tissue MS/MS are often required to confirm metabolite identities. Figure 2 describes these challenges which are compounded by the fact that comprehensive, MSI-specific spectral libraries remain limited and resource-intensive particularly for small polar metabolites and isomeric species, in contrast to the resources available for bulk metabolomics.

MSI data are inherently semi-quantitative and subject to pixel-to-pixel variability arising from matrix effects, heterogeneous matrix-analyte co-crystallization, sample composition, and local ion suppression. As a result, ion intensity maps reflect relative abundance rather than absolute concentration, and signal intensities are not directly comparable across metabolites or even across regions for different metabolites (Tobias and Hummon, 2020). Strategies such as on-tissue application of stable isotope–labeled internal standards, calibration curves with tissue-mimetic models can improve quantitative interpretation by normalizing local ionization efficiency, but these approaches are labor-intensive and typically limited to a small number of targeted metabolites. Often, comprehensive sets of internal standards are not available, and common normalization approaches like total ion current (TIC), root mean square (RMS), or to specific matrix peaks are used each with distinct assumptions and limitations discussed in literature (Baquer et al, 2023). This is critical for image presentation and can significantly affect the interpretation of MSI data.

These quantification constraints constitute potential boundaries for biological interpretation of imaging datasets. As an example, spatial intensity differences in ion images do not necessarily reflect true concentration gradients if experimental parameters were not optimized and validated. More specifically, pixel-to-pixel variability arising from heterogeneous matrix crystallization, mass errors, and local ion suppression complicates statistical comparisons between anatomical regions; observed differences may reflect technical artifacts rather than biology. Also, direct comparison of signal intensities across different metabolites is invalid, as ionization efficiencies vary by orders of magnitude depending on chemical class, functional groups, and local tissue environment. Consequently, claims such as “metabolite A is more abundant than metabolite B in region X” or “metabolite C shows a 2-fold gradient across the tissue” cannot be reliably made from MSI data alone without orthogonal validation (Fig. 2). Thus, biological claims should rather focus on relative spatial distributions, co-localization patterns, metabolite ratios, and qualitative differences between experimental groups, with caution about quantitative statements unless proper standard calibration or validation has been performed.

In the case of stable isotope tracer imaging, interpreting ion images requires normalization to the actual circulating tracer levels; it is important to measure how much of the tracer reached the tissue globally. Often, an aliquot of blood plasma is collected during the time course and at the time of sacrifice to measure circulating labeled substrate or its metabolites by LC–MS (Wang et al, 2022b).

Data dimensionality further complicates interpretation. Each pixel in an MSI experiment contains a full mass spectrum with thousands of features, often across hundreds to thousands of pixels per section. When combined with additional spatial omics layers, such as transcriptomics or proteomics, data complexity rapidly exceeds what can be interpreted manually (Baquer et al, 2023; Saw et al, 2025). Moreover, metabolites are chemically labile and may not colocalize with their sites of biosynthesis or metabolism, further complicating straightforward spatial interpretation. To address this complexity, computational and machine learning approaches have become essential (Wadie et al, 2024).

Finally, biological context is indispensable for interpretation, and metabolite images in isolation can be misleading, for example, elevated lactate, may reflect increased glycolysis, hypoxia, or simply higher local cell density. Multimodal imaging and spatial validation approaches discussed earlier should be an integral part of the workflow.

## Outlook

Spatial metabolomics is rapidly maturing from a niche technology to a central component of the spatial biology toolkit (Alexandrov, 2023). A notable trend is its convergence with other spatial omics methods to build a comprehensive single-cell atlases of tissue function (Osetrova et al, 2024). Beyond research applications, a compelling vision for translating spatial metabolomics is to improve human health with precision diagnostics and personalized metabolic medicine. To reach this goal, the challenges discussed here related to technology should be carefully addressed: improving throughput, data quality and interpretability, MSI accessibility, developing robust computational pipelines, and validating them in clinical settings. Considering these aspects, the ultimate promise of mapping metabolism at cellular resolution will not only expand our fundamental knowledge but also provide new paths to treat diseases by targeting the right metabolism in the right place.

However, several fundamental barriers remain to be addressed before widespread translational utilization of spatial metabolomics tools. A major current bottleneck is the absence of standardized, MSI-specific spectral libraries for small metabolites for confident metabolite identification (Smith et al, 2025) and the limited quantitative capability of MSI data compared to bulk metabolomics. Inter-laboratory and cross-platform reproducibility, standardization guidelines, and harmonization of protocols need to be addressed as a priority. These challenges are further compounded by limited throughput, costs, and required specialized expertise. Resource and time considerations distinguish spatial metabolomics from bulk approaches and constrain its broader adoption.

Despite these barriers, consensus data analysis pipelines and validation frameworks make considerable progress and improve quantitative capabilities of spatial metabolomics methods. Once these challenges are addressed to the extent where mapping metabolism at cellular resolution is reliable, it will not only expand our fundamental knowledge but also provide new therapeutic paths by targeting the metabolism in the right place.

### Key considerations

- Spatial metabolomic studies should be hypothesis-driven and guided by appropriate controls and spatial context.
- Consider metabolite coverage, stability, and quantitative limitations, which are strongly influenced by sample preparation, pretreatments, and data acquisition parameters.
- Metabolite maps require biological context through co-registration with histology and molecular markers, combined with computational analysis to extract meaningful biological insights.

## Supplementary information

Table EV1

Table EV2

Peer Review File
