# Supplementary material for: Spatial metabolomics: design, pitfalls and data interpretation
Source: EMBO J. 2026 May 2;45(12):4007–13. doi: 10.1038/s44318-026-00797-x (PMC13270173; doi:10.1038/s44318-026-00797-x)
Supplement: Supplementary file 1 — Table EV1 [file 44318_2026_797_MOESM1_ESM.docx]

***Table EV1:*** List of different spatial metabolomics technologies with their description and applications.

| **Technique / Modality** | **Principle** | **Sample Prep** | **Spatial Resolution** | **Molecular Coverage** | **Strengths** | **Limitations** | **Typical Applications** |
| --- | --- | --- | --- | --- | --- | --- | --- |
| **MALDI-MSI** (Matrix-Assisted Laser Desorption Ionization) | Laser ablation of matrix-coated tissue, matrix assists ionization | Fresh-frozen, matrix coating (e.g., CHCA, DHB, 9-AA) on conductive glass slides | 5–50 µm (single-cell possible with advanced methods) | Small metabolites → lipids → peptides | Broad coverage, high sensitivity, established workflows, tandem MS possible | Matrix background in low *m/z*; requires vacuum; analyte delocalization risk from wet matrix | Tissue metabolomics, lipidomics, drug distribution, pathology mapping |
| **MALDI-2** (Post-ionization MALDI) | Secondary laser post-ionizes neutrals in plume | Same as MALDI | 5–20 µm | Improves low-abundance and low-mass metabolite coverage | Boosts sensitivity (10–100x); better for lipids, sterols, sugars | Needs specialized laser setup; adoption still limited | Lipidomics, and recent developments in low-abundance signaling metabolites |
| **DESI-MSI** (Desorption Electrospray Ionization) | Charged solvent droplets desorb/ionize surface analytes | Fresh-frozen section; minimal prep; solvent spray (MeOH/H₂O etc.) | ~35–100 µm (Nano-DESI: ~ 20 µm) | Small metabolites, lipids, drugs; emerging for proteins | Minimum, ambient acquisition, no matrix; clean low *m/z* spectra, possible on fresh tissue | Lower throughput; sensitive to solvent composition & surface | Clinical tissue and tumor diagnosis, drug/metabolite imaging, lipidomics |
| **SIMS / NanoSIMS** (Secondary Ion MS) | Focused ion beam sputters secondary ions from surface | Dry/freeze-dried tissue on conductive substrate; often vacuum | 50 nm – 2 µm | Mostly small molecules (fragments <500 Da), elements, isotopes | Unparalleled spatial resolution (subcellular, organelle-level); isotope tracing; element mapping | Limited molecular coverage (fragmentation); destructive; specialized instrumentation | Subcellular metabolite mapping, isotope tracing, elemental imaging |
| **LAESI / MALDESI** (Laser Ablation Electrospray Ionization) | IR or UV laser ablates microplume, post-ionized by ESI | Fresh/frozen tissue; minimal prep | ~100 µm | Polar metabolites, lipids, drugs | Ambient, matrix-free; depth profiling possible | Lower resolution; fewer commercial platforms | Plant tissues, water-rich organs, drug/metabolite imaging |
| **LDI/NIMS** (Nanostructure-assisted LDI) | Laser desorption from nanostructured surface (no organic matrix) | Special substrates (e.g., porous silicon, nanowires) | ~10–20 µm | Low-mass metabolites (<500 Da), small molecules | Matrix-free clean spectra in low-m/z range | Limited adoption due to instrumental complexity, substrate preparation required | Imaging small metabolites, drugs, toxins |
| **LESA Imaging** (Liquid Extraction Surface Analysis) | Microdroplet extracts local analytes, then analyzed by MS | Fresh/frozen, stable mounting | ~500–1000 µm (low) | Broad — metabolites, lipids, peptides, proteins | Very high sensitivity; allows LC-MS coupling | Very coarse resolution; slow throughput | Targeted regional metabolomics, drug quantitation |
| **Imaging Mass Cytometry (MIBI / CyTOF Imaging)** | Metal-tagged antibodies detected by ion beam | Fixed tissue, antibody labeling | ~0.5–1 µm | Proteins, cell-surface markers | Multiplexed protein/cell-type mapping | Not metabolomics; antibody availability limits scope | Cell phenotyping, tumor immune microenvironment |
| **Laser Microdissection (LMD / LCM)** | UV laser cuts out regions of interest from same or consecutive tissue sections | Fresh-frozen, thin section on PEN/ITO membrane | ~10 -100 µm (region of interest defined) | Not imaging; relies on LC-MS analysis after extraction | Enables spatially precise metabolomics/proteomics; validates MSI regions | Destructive; no direct imaging; labor and resource intensive | ROI validation, targeted metabolomics after MSI |
| **On-Tissue Chemical Derivatization (OTCD) and Glycan MALDI-MS** | Chemical spray reacts with functional groups of metabolites in situ before MSI | Fresh-frozen, derivatization reagents applied | Not applicable | Improves detectability of carbonyls, amines, sugars, glycans | Expands coverage, enhances sensitivity, differentiates isomers | Risk of delocalization; reagent optimization required | Carbohydrate, amino acid, glycan imaging, and isomer separation |
