# Supplementary material for: Spatial metabolomics: design, pitfalls and data interpretation
Source: EMBO J. 2026 May 2;45(12):4007–13. doi: 10.1038/s44318-026-00797-x (PMC13270173; doi:10.1038/s44318-026-00797-x)
Supplement: Supplementary file 2 — Table EV2 [file 44318_2026_797_MOESM2_ESM.docx]

***Table EV2:*** List of available sample preparation and preservation methods for spatial metabolomics analysis with their principle, advantages and limitations for MSI.

| **Method** | **Principle** | **Advantages** | **Limitations / Artefacts** | **Suitability for MSI** |
| --- | --- | --- | --- | --- |
| **Snap-freezing** (Liquid N₂ / isopentane) | Rapid temperature drop quenches enzymatic activity | Preserves native metabolite distributions; compatible with untargeted MSI | Requires speed and expertise; ice crystal artefacts if slow | Gold standard for tissue preservation |
| **Cryo-embedding** (CMC, gelatin) | Stable mechanical support for cryosectioning | Enables thin, reproducible sections | MS background; potential diffusion if not fully frozen | Need to optimize protocols with background |
| **Focused microwave irradiation** (FMW) | Rapid heating inactivates enzymes | Stabilizes labile metabolites | Thermal gradients; limited tissue penetration | Tissue-dependent |
| **In situ microwave fixation** | Instantaneous enzyme inactivation *in vivo* | Minimizes post-harvest artefacts | Specialized equipment; limited availability | Emerging, potential with further adoption and validation |
| **Funnel-freezing / in situ freezing** | Rapid freezing under maintained perfusion | Superior preservation of brain metabolism | Technically complex, expertise | High relevant for brain metabolism |
| **Chemical fixation (FFPE)** | Protein crosslinking / precipitation | Excellent morphology | Metabolite loss, modification, ion suppression | Generally incompatible |
| **Saline perfusion** | Removes blood prior to freezing | Reduces blood-derived signal contamination | washout effects | Need optimization, relevant to vascular tissues |
