# Supplementary material for: Spatial metabolomics: design, pitfalls and data interpretation
Source: EMBO J. 2026 May 2;45(12):4007–13. doi: 10.1038/s44318-026-00797-x (PMC13270173; doi:10.1038/s44318-026-00797-x)
Supplement: Supplementary file 3 — Peer Review File [file 44318_2026_797_MOESM3_ESM.pdf]

THE EMBO JOURNAL

Spatial Metabolomics: Design, Pitfalls and Data Interpretation

Prasad Phapale

Corresponding author: Prasad Phapale (prasad.phapale@envs.au.dk)

Review Timeline:

Submission Date:	8th Feb 26
Editorial Decision:	11th Mar 26
Revision Received:	8th Apr 26
Editorial Decision:	14th Apr 26
Revision Received:	15th Apr 26
Accepted:	21st Apr 26

Editor: Daniel Klimmeck

Transaction Report:

(Note: With the exception of the correction of typographical or spelling errors that could be a source of ambiguity, letters and reports are not edited. Depending on transfer agreements, referee reports obtained elsewhere may or may not be included in this compilation. Referee reports are anonymous unless the Referee chooses to sign their reports.)

1st Editorial Decision **11th Mar 2026**

Dear Dr Phapale,

Thank you again for sending us your commentary article for the metabolism advice series. As mentioned, we have asked two dedicated spatial metabolomics experts to assess your manuscript, and in the meantime got feedback from both of them, which I enclose below.

As you will see, the colleagues much appreciate the perspective piece and find it timely and worth publishing. They also provide constructive feedback on how to further improve it by additional discussion of relevant aspects, including remaining limitations and ambiguities of the methodology, choice of experimental design and data interpretation workflows, broadening and illustrating conceptual considerations and potential use-cases, as well as better annotating technical modalities available.

With respect to the artwork, please let me know about your views about the referee's point. You might consider adjustments and enhancements by adding 1-2 additional figure panels or figures. In any case, a preliminary next version of the sketch will be sufficient as a basis for final rendering by our scientific illustration team.

I hope you will find the comments helpful. I am sure that an amended version incorporating the suggestions made by the referees will be highly noted and appreciated. I would thus like to invite you to submit such a revised version using the link enclosed below.

Please let me know in case I can be of any help with this.

Best regards,

Daniel Klimmeck

Daniel Klimmeck, PhD
Senior Editor
The EMBO Journal

Further information is available in our Guide For Authors: https://www.embopress.org/page/journal/14602075/authorguide

----

Referee #1:

This Perspective aims to provide an overview of experimental design, sample preparation, and interpretative considerations in spatial metabolomics, particularly mass spectrometry imaging (MSI). The article highlights the promise of spatially resolved metabolic measurements to reveal intra-tissue heterogeneity and discusses practical aspects ranging from tissue handling to multimodal integration and computational analysis.
The topic is timely and relevant. Spatial metabolomics is expanding rapidly, and guidance documents are valuable for investigators entering the field. However, in its current form the manuscript reads largely as a technical checklist rather than a critical conceptual framework. For a Perspective in EMBO Journal, the article would benefit from deeper analysis of interpretative limitations, chemical specificity, and sources of analytical artefact that directly impact biological conclusions.

Major Comments
1. The manuscript underemphasizes chemical specificity and risks of misannotation
The text acknowledges that MSI experiments are often performed in MS1 mode and that metabolite identification is limited without targeted validation.

However, this issue is presented as a technical inconvenience rather than a central limitation of spatial metabolomics.
In contrast to bulk LC-MS workflows, MSI datasets frequently lack:
• Chromatographic separation
• Orthogonal fragmentation evidence
• Quantitative calibration
• Robust control of ion chemistry
This combination makes structural assignment intrinsically ambiguous. A Perspective should explicitly discuss that many MSI-derived "metabolite maps" represent putative ion distributions, not confirmed metabolite localization. Without this clarification, readers may overinterpret spatial patterns biologically.

2. Insufficient discussion of ion chemistry-driven artefacts (a major omission)
The manuscript briefly notes that adduct formation and in-source fragmentation can lead to misannotation
, but this issue deserves far more critical treatment.
There is now substantial evidence across electrospray and laser-based ionization methods that:
• Gas-phase and droplet-phase reactions can generate new ions not present in the tissue.
• In-source fragmentation can produce abundant low-mass species that are misassigned as endogenous metabolites.
• Matrix and surface chemistry strongly influence apparent metabolite distributions.
Because spatial metabolomics lacks chromatographic validation, these phenomena may disproportionately affect MSI interpretations compared to LC-MS metabolomics.
A Perspective intended to guide experimental design should explicitly address:
• Chemical artefact generation during desorption/ionization.
• The need for orthogonal validation (LC-MS, isotope tracing, or standards).
• The risk that spatial heterogeneity partly reflects ionization physics rather than biology.
At present, the manuscript treats MSI largely as a measurement problem rather than also a reaction environment.

3. The article focuses heavily on workflow logistics but lacks a unifying conceptual model
Large portions of the manuscript describe sectioning, freezing, embedding media, and handling procedures. While useful, these resemble a methods review rather than an EMBO-style Perspective.
What is missing is a framework addressing:
• What spatial metabolomics can uniquely answer vs. what it cannot.
• How spatial resolution trades off against molecular certainty.
• When bulk metabolomics is actually more appropriate.
• The hierarchy of confidence (detection → annotation → quantification → flux inference).
A conceptual diagram of levels of interpretability would strengthen the manuscript far more than procedural detail.

4. Quantification limitations are acknowledged but not critically explored
The authors state that MSI data are "inherently semi-quantitative" and subject to matrix and ion suppression effects.
This is an essential point but is not followed by discussion of how this constrains biological claims.
For example:
• Spatial intensity differences cannot be assumed to reflect concentration gradients.
• Pixel-to-pixel variability complicates statistical inference.
• Cross-metabolite comparisons are often invalid.
The Perspective should guide readers toward interpretation boundaries, not just technical mitigation strategies.

5. The current narrative is optimistic but insufficiently critical for a field-shaping Perspective
The manuscript concludes with a vision of spatial metabolomics enabling precision diagnostics and personalized metabolic medicine.

Such claims require a more balanced discussion of current barriers:
• Lack of standardized spectral libraries for MSI
• Reproducibility across platforms and laboratories
• Limited validation pipelines
• Data analysis pipelines still lacking consensus
Without this balance, the Perspective risks appearing promotional rather than analytical.

Referee #2:

Phapale provides a perspective on mass spectrometry imaging (MSI) as a technology to interrogate metabolic heterogeneity in biological samples. This work discusses MSI applications, highlights certain pitfalls and is likely to be of interest to the general scientific community. However, it is a brief introduction of the technology and does not exactly provide the critical guide to design experiments/interpret data for early adopters of the field it aspires. In that context, I suggest the following improvements:

Experimental design

1. Discuss how MSI workflows do not allow selective enrichment of certain metabolites through solvent-based extraction, thereby complicating the detection of lowly abundant metabolites.

2. The manuscript focuses on MALDI MSI. It is critical to provide a detailed overview of the different modalities that are available (e.g. DESI-MSI, LD-REIMS,...) and how platform choice might affect metabolite coverage. Also, a section should be included on high-resolution (subcellular) imaging (e.g. GCIB-SIMS, OrbiSIMS, NanoSIMS) and how these can be used to study metabolite compartmentalisation, enzyme complexes etc.

3. The need for maximizing tissues on a single slide is mentioned but a more careful explanation of batch effects and how to minimize/recognize these is required.

4. The statistical methods to determine sample size need to be discussed, n=3-5 is arbitrary and very context dependent.

5. Normalization approaches to imaging mass spectral data need to be discussed

Sample collection and quenching

1. Caveats/benefits of freezing in liquid nitrogen/dry ice should be discussed as well as other commonly used cryogens (e.g. liquid nitrogen/isopentane).

2. More discussion on the embedding strategy and criteria for mounting media selection (e.g. the need for a supportive scaffold, correct viscosity, minimal interference with MSI, no adverse effects on histology, cost,...). Caveats of carboxymethylcellulose need to be discussed (e.g. ion suppression, salt adducts) and other options should be highlighted (e.g. HPMC/PVP-enriched hydrogels).

3. While imaging FFPE-fixed tissues comes with significant complications, a lot of research has been done to maximally extract useful information from such samples, which are often more readily available with large historic collections banked. This needs to be discussed.

Finally, it would be informative to highlight cost and time as compared to traditional metabolomics, and hardly any information is provided on data analysis and current views on integration with other omics technologies.

Rebuttal to Reviewers Comments:

Referee #1:

This Perspective aims to provide an overview of experimental design, sample preparation, and interpretative considerations in spatial metabolomics, particularly mass spectrometry imaging (MSI). The article highlights the promise of spatially resolved metabolic measurements to reveal intra-tissue heterogeneity and discusses practical aspects ranging from tissue handling to multimodal integration and computational analysis.

The topic is timely and relevant. Spatial metabolomics is expanding rapidly, and guidance documents are valuable for investigators entering the field. However, in its current form the manuscript reads largely as a technical checklist rather than a critical conceptual framework. For a Perspective in EMBO Journal, the article would benefit from deeper analysis of interpretative limitations, chemical specificity, and sources of analytical artefact that directly impact biological conclusions.

→ I thank the reviewer for appreciating the article and constructive comments on improvement. Several changes are not made with additional figures and tables, text improvement addressing each comment. I hope this will improve the depth of the article while critically evaluating limitations and nuances of spatial metabolomics.

Major Comments

1. The manuscript underemphasizes chemical specificity and risks of mis annotation

The text acknowledges that MSI experiments are often performed in MS1 mode and that metabolite identification is limited without targeted validation.

However, this issue is presented as a technical inconvenience rather than a central limitation of spatial metabolomics.

In contrast to bulk LC-MS workflows, MSI datasets frequently lack:

• Chromatographic separation
• Orthogonal fragmentation evidence
• Quantitative calibration
• Robust control of ion chemistry

This combination makes structural assignment intrinsically ambiguous. A Perspective should explicitly discuss that many MSI-derived "metabolite maps" represent putative ion distributions, not confirmed metabolite localization. Without this clarification, readers may overinterpret spatial patterns biologically.

→ I thank reviewer for detailed context about the metabolite identification and agree with this critical issue. There has been increasing awareness about the issue which we started with development of METASPACE annotation platform since 2017 and realizing its limitations of even after FDR controlled MS1 annotations.

Now I have added additional **Schematic (Figure 2)** describing integrated strategy annotation and confidence level metabolite identification in the context of MSI data this Figure and related description will provide conceptual framework new scientist to navigate nuances and trade-off of metabolite identification in MS imaging datasets.

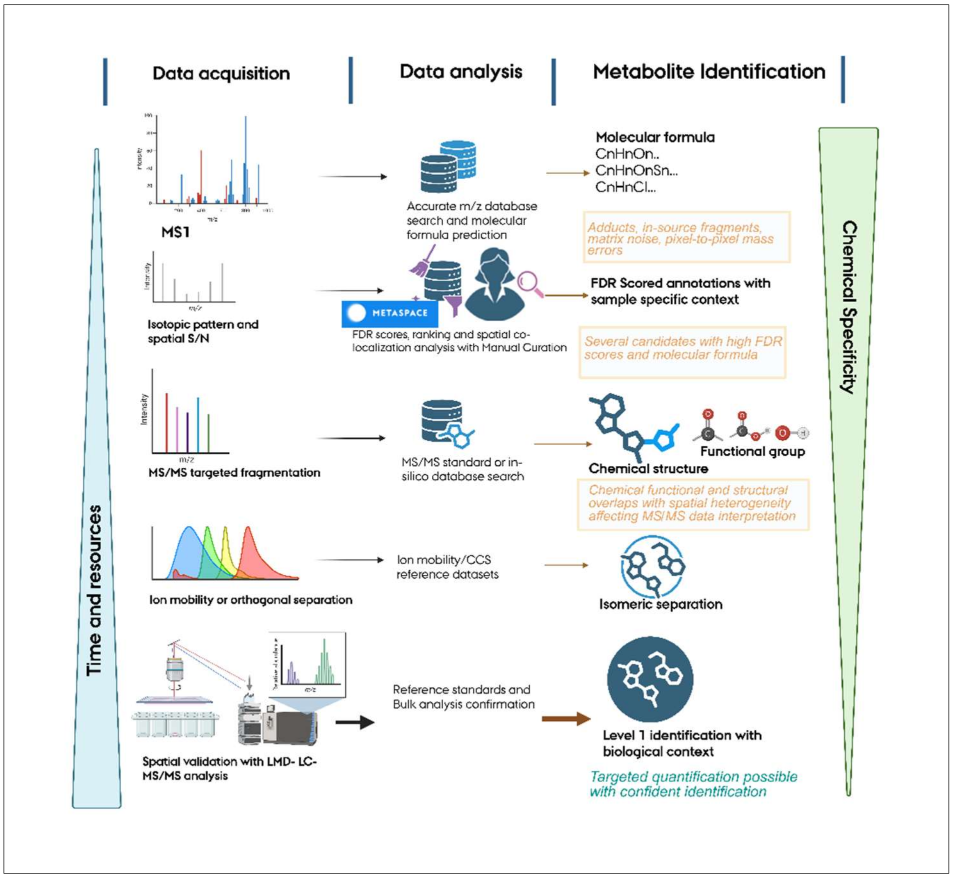

Figure 2: Integrated MS imaging strategy for metabolite identification: *data acquisition approaches, annotation platforms, and validation methods with corresponding trade-off of time/resource requirements with identification confidence and chemical specificity achieved.*

2. Insufficient discussion of ion chemistry-driven artefacts (a major omission)
The manuscript briefly notes that adduct formation and in-source fragmentation can lead to misannotation, but this issue deserves far more critical treatment.

There is now substantial evidence across electrospray and laser-based ionization methods that:
• Gas-phase and droplet-phase reactions can generate new ions not present in the tissue.
• In-source fragmentation can produce abundant low-mass species that are misassigned as endogenous metabolites.
• Matrix and surface chemistry strongly influence apparent metabolite distributions.
Because spatial metabolomics lacks chromatographic validation, these phenomena may disproportionately affect MSI interpretations compared to LC-MS metabolomics.
A Perspective intended to guide experimental design should explicitly address:
• Chemical artefact generation during desorption/ionization.
• The need for orthogonal validation (LC-MS, isotope tracing, or standards).
• The risk that spatial heterogeneity partly reflects ionization physics rather than biology.
At present, the manuscript treats MSI largely as a measurement problem rather than also a reaction environment.

→ I agree with reviewer's comment, and we face these challenges on daily basis with MS imaging datasets. ***Figure 2*** (in orange boxes) specifically addressed these issues for each stage of data interpretation and its potential resolution at next stage and ultimately with spatial heterogeneity. Although discussion is added in the text, addressing each issue in more detail will be too technical and beyond the scope of this perspective.

3. The article focuses heavily on workflow logistics but lacks a unifying conceptual model
Large portions of the manuscript describe sectioning, freezing, embedding media, and handling procedures. While useful, these resemble a methods review rather than an EMBO-style Perspective.
What is missing is a framework addressing:
• What spatial metabolomics can uniquely answer vs. what it cannot.
• How spatial resolution trades off against molecular certainty.
• When bulk metabolomics is actually more appropriate.
• The hierarchy of confidence (detection → annotation → quantification → flux inference).
A conceptual diagram of levels of interpretability would strengthen the manuscript far more than procedural detail.

→ I agree with reviewer, while this is partly addressed in Figure 1 and relevant text is now expanded to showcase the specific examples of spatial metabolomics in the section '*Spatial Metabolomics Illuminates Metabolic Heterogeneity*'. Again, the Figure 2 specifically addresses the reviewer concern about 'hierarchy of confidence'.

4. Quantification limitations are acknowledged but not critically explored

The authors state that MSI data are "inherently semi-quantitative" and subject to matrix and ion suppression effects.
This is an essential point but is not followed by discussion of how this constrains biological claims.
For example:

- Spatial intensity differences cannot be assumed to reflect concentration gradients.
- Pixel-to-pixel variability complicates statistical inference.
- Cross-metabolite comparisons are often invalid.

The Perspective should guide readers toward interpretation boundaries, not just technical mitigation strategies.

→ This is valuable comments and the points mentioned by in the reviewer is valuable. I have now expanded the discussion with following text:

…… ..limited to a small number of targeted metabolites. 'These quantification constraints may limit critical boundaries on biological interpretation of imaging datasets. Spatial intensity differences in ion images do not necessarily reflect true concentration gradients if experimental parameters were not optimized and validated. For example, pixel-to-pixel variability arising from heterogeneous matrix crystallization, mass errors and local ion suppression complicates statistical comparisons between anatomical regions; observed differences may reflect technical artifacts rather than biology. Also, direct comparison of signal intensities across different metabolites is invalid, as ionization efficiencies vary by orders of magnitude depending on chemical class, functional groups, and local tissue environment. Consequently, claims such as "metabolite A is more abundant than metabolite B in region X" or "metabolite C shows a 2-fold gradient across the tissue" cannot be reliably made from MSI data alone without orthogonal validation (Figure 2). Thus, biological claims should focus on relative spatial distributions, co-localization patterns, metabolite ratios, and qualitative differences between experimental groups, with caution about quantitative statements unless proper standard calibration or validation has been performed.'

5. The current narrative is optimistic but insufficiently critical for a field-shaping Perspective

The manuscript concludes with a vision of spatial metabolomics enabling precision diagnostics and personalized metabolic medicine.
Such claims require a more balanced discussion of current barriers:

- Lack of standardized spectral libraries for MSI
- Reproducibility across platforms and laboratories
- Limited validation pipelines
- Data analysis pipelines still lacking consensus

Without this balance, the Perspective risks appearing promotional rather than analytical.

→ I agree with reviewers critical points and now each mentioned points is now added into discussion and Outlook part of the manuscript with following text:.

'However, several fundamental barriers remain to be addressed before widespread translational utilization of spatial metabolomics tools. Major bottleneck is absence of standardized, MSI-specific spectral libraries for small metabolites for confident metabolite identification and limited quantitative capability of MSI data compared to bulk metabolomics. Inter-laboratory and cross-platform reproducibility, standardization guidelines and harmonization of protocols need to address with urgency. These challenges are further compounded by limited throughput, costs, and required specialized expertise. Despite these barriers, consensus data analysis pipelines and validation frameworks make considerable progress and improve quantitative capabilities of spatial metabolomics methods. Once these challenges are addressed to the extent where mapping metabolism at cellular resolution is reliable, it will not only expand our fundamental knowledge but also provide new treatment paths to diseases by targeting the right metabolism in the right place.'

Referee #2:

Phapale provides a perspective on mass spectrometry imaging (MSI) as a technology to interrogate metabolic heterogeneity in biological samples. This work discusses MSI applications, highlights certain pitfalls and is likely to be of interest to the general scientific community. However, it is a brief introduction of the technology and does not exactly provide the critical guide to design experiments/interpret data for early adopters of the field it aspires. In that context, I suggest the following improvements:

Experimental design

1. Discuss how MSI workflows do not allow selective enrichment of certain metabolites through solvent-based extraction, thereby complicating the detection of lowly abundant metabolites.

→ I thank the reviewer for this important and critical point which is now addressed under section of *Sample pre-treatments to enhance metabolite imaging* with following new text:

'In contrast to solvent based metabolites extraction for bulk analysis, MSI requires metabolites to remain in situ within tissue sections, limiting enrichment of metabolites. Consequently, low-abundance metabolites, including many signaling molecules, intermediates, and regulatory metabolites are often undetectable or suppressed by more abundant lipids and matrix ions, particularly in the low m/z range (<500 Da). While OTCD and matrix selection can partially mitigate this limitation for specific compound

classes, MSI inherently favors detection of abundant metabolites and lipids with limited coverage of the low-abundance metabolome compared to bulk approaches.'

2. The manuscript focuses on MALDI MSI. It is critical to provide a detailed overview of the different modalities that are available (e.g. DESI-MSI, LD-REIMS,...) and how platform choice might affect metabolite coverage. Also, a section should be included on high-resolution (subcellular) imaging (e.g. GCIB-SIMS, OrbiSIMS, NanoSIMS) and how these can be used to study metabolite compartmentalisation, enzyme complexes etc.

→I agree with his missing part and now an additional **Supplementary Table 1**: List of different spatial metabolomics technologies with their description and applications. The table details all major techniques and modalities with their operation Principle, Sample Prep methods, Spatial Resolution, Molecular Coverage possible, Strengths and Limitations with Typical Applications.

3. The need for maximizing tissues on a single slide is mentioned but a more careful explanation of batch effects and how to minimize/recognize these is required.

→ I agree with the quality control aspects which are bottleneck and main pain point of MSI data reliability. Several discussion points are now added in the Experimental Design and conclusion section to address this point.

4. The statistical methods to determine sample size need to be discussed, n=3-5 is arbitrary and very context dependent.

→ I thank reviewer for highlighting this gap and more discussion about sample number is now added under Experimental design section as follows:

'However, determining adequate sample size for spatial metabolomics is more complex than for bulk analyses. The appropriate n depends on expected group effect size (within sample and among samples), spatial heterogeneity within and between individual tissue sections (same tissue different depth), the number of metabolites and regions being compared as well as available resources. Whenever possible, pilot studies are recommended using sample subsets to inform power calculations by estimating within-group variance and effect sizes for key metabolites or regions.'

5. Normalization approaches to imaging mass spectral data need to be discussed

→ Brief discussion about normalization methods is now added with a reference within section of Challenges in Data Interpretation.

Sample collection and quenching

1. Caveats/benefits of freezing in liquid nitrogen/dry ice should be discussed as well as other commonly used cryogens (e.g. liquid nitrogen/isopentane).

→I agree with his missing technical part and now an additional **Supplementary Table 2** with List of available sample preparation and preservation methods for spatial metabolomics analysis with their principle, advantages and limitations for MSI.

2. More discussion on the embedding strategy and criteria for mounting media selection (e.g. the need for a supportive scaffold, correct viscosity, minimal interference with MSI, no adverse effects on histology, cost,...). Caveats of carboxymethylcellulose need to be discussed (e.g. ion suppression, salt adducts) and other options should be highlighted (e.g. HPMC/PVP-enriched hydrogels).

→ The discussion on the part is expanded with addition **Supplementary Table 2.**

3. While imaging FFPE-fixed tissues comes with significant complications, a lot of research has been done to maximally extract useful information from such samples, which are often more readily available with large historic collections banked. This needs to be discussed.

→This is valuable mention and I have expanded the FFPE discussion with following text:

'FFPE tissue represents an invaluable vast resource of archival collections over decades of clinical specimens linked to patient outcomes, diagnostic data, and longitudinal follow-up. Accessing this biobank material would enable retrospective spatial metabolomics studies that are otherwise difficult with fresh-frozen tissue requirements. Despite challenges, progress has been made in extracting metabolic information from FFPE samples using dewaxing methods, solvent treatments and some studies targeted on stable lipid analysis since most polar metabolites are lost in treatments(Denti et al, 2022).'

Finally, it would be informative to highlight cost and time as compared to traditional metabolomics, and hardly any information is provided on data analysis and current views on integration with other omics technologies.

→I thank reviewer for highlighting this interesting but missing aspect in the earlier version which is now updated. Although I am hesitant to put exact cost calculations on the time constraints and detailed in the Experimental design and Outlook section.

Dear Prasad,

Thank you for your patience with the experts' feedback on your revised Comment manuscript. We have now received additional comments by the two colleagues. As you will see they state that the commentary has been substantially improved by your amendments, and they recommend publication, pending minor remaining changes.

Hence, I am happy to share that your Comment is provisionally accepted for publication at the EMBO Journal.

Please revisit the additional remarks by expert #2 and adjust the text and discussion where appropriate. I will myself also go again through the manuscript text and suggest final minor changes and edits shortly.

Also, I kindly ask you to consider formatting requests as to the list enclosed below.

I look forward to your last revision and moving ahead with formal acceptance and publication of this commentary shortly.

Best wishes,

Daniel

Daniel Klimmeck, PhD
Senior Editor | The EMBO Journal
d.klimmeck@embojournal.org

Instructions for preparing your revised manuscript:

>> please check callouts: Fig 1 is currently not called out.

>> Figures: remove from manuscript text and upload as separate figure files, legends should be moved to the end of the manuscript text.

>> Tables: rename the suppl. tables to Table EV1 and Table EV2 and upload as two separate files.

Further information is available in our Guide For Authors: https://www.embopress.org/page/journal/14602075/authorguide

----

Referee #1:

The paper is now suitable for publication.

Referee #2:

My main concerns have been adequately addressed in this revised version. I would recommend to change the wording of

the

following sentences to improve accuracy:

* 'As in bulk metabolomics, biological replication remains critical; multiple independent samples per condition (typically n = 3-5) are required to ensure that observed spatial patterns are reproducible and statistically meaningful.'

As mentioned previously, n=3-5 is arbitrary, highly depends on each experimental question and is typically not sufficient to resolve differences using MSI, particularly in human samples. Therefore I suggest removing n=3-5 as a suggested sample size.

* 'Tumor tissues present the most complex challenge' to 'Analysing tumour tissues often comes with added complexity' or similar.

* 'This is critical part in image presentation and can significantly affect the visual interpretation of MSI data.'

I suggest removing 'visual' here. Normalization is critical for MSI data interpretation in its entirety.

Finally, the manuscript needs proofreading to address some typo's and grammatical errors.

The authors addressed the remaining formatting issues.

Dear Dr Phapale,

Thank you for sending us the updated final version of the commentary article.

I am pleased to inform you that your manuscript has been accepted for publication in the EMBO Journal.

Your manuscript will be processed for publication by EMBO Press. It will be copy edited and you will receive page proofs prior to publication. Please note that you will be contacted by Springer Nature Author Services to complete licensing and payment information. As this is invited front-half content, OA charges applicable to this article will be covered.

We will proceed with transfer of the manuscript files to production shortly.

If you have any questions, please do not hesitate to contact me.

Thank you again for your kind contribution to The EMBO Journal series, which is much appreciated.

with
Best regards,

Daniel Klimmeck

Daniel Klimmeck, PhD
Senior Editor
The EMBO Journal

*****
Please note that it is The EMBO Journal policy for the transcript of the editorial process (containing referee reports and your response letters) to be published as an online supplement to each paper. If you should prefer removal of any referee-only figures included in the point-by-point response(s), e.g. because they may still be used for future publication or because they have been reproduced from published work by others, please do let us know immediately via response email.
More information is available here: https://link.springer.com/partners/embo-press/editorial-policies#Peer%20review

** Click here to be directed to your login page: https://emboj.msubmit.net
